# TBCK Influences Cell Proliferation, Cell Size and mTOR Signaling Pathway

**DOI:** 10.1371/journal.pone.0071349

**Published:** 2013-08-19

**Authors:** Yueli Liu, Xiaoyi Yan, Tianhua Zhou

**Affiliations:** Department of Cell Biology and Program in Molecular Cell Biology, School of Medicine, Zhejiang University, Hangzhou, Zhejiang, China; Sudbury Regional Hospital, Canada

## Abstract

Mammalian target of rapamycin (mTOR) is a central regulator for both cell proliferation and cell growth; however, little is known about the regulation of mTOR expression at the transcriptional level. Here, we provide evidences that a conserved human protein TBCK (TBC1 domain containing kinase) is involved in the regulation of mTOR signaling pathway. Depletion of TBCK significantly inhibits cell proliferation, reduces cell size, and disrupts the organization of actin, but not microtubule. Knockdown of TBCK induces a significant decrease in the protein levels of components of mTOR complex (mTORC), and suppresses the activity of mTOR signaling, but not MAPK or PDK1/Akt pathway. Further results show that TBCK influences the expression of mTORC components at the transcriptional level. Thus, these data suggest that TBCK may play an important role in cell proliferation, cell growth and actin organization possibly by modulating mTOR pathway.

## Introduction

Cell proliferation and cell growth (increased in cell size and cell mass) are distinct, but coupled processes that are essential for development of an organ and whole organism. To achieve the optimal size of cell, organ, or organisms, cell growth and cell proliferation must be tightly coordinated [1]; however, the underlying mechanism is poorly understood. Emerging data show that mTOR (mammalian target of rapamycin or mechanistic target of rapamycin) regulates both cell proliferation and cell growth in various species from yeast to human and thus is believed as a key player in these biological processes [2]–[5].

In general, mTOR interacts with several proteins to form two distinct complexes named mTOR complex 1 (mTORC1) and mTOR complex 2 (mTORC2) [6]. Both complexes share mTOR, mammalian lethal with SEC13 protein 8/G-protein β-subunit like protein (mLST8/GβL), DEP domain containing mTOR-interacting protein (DEPTOR), and the Tit1/Tel2 complex [6]–[9]. In addition, mTORC1 has regulatory-associated protein of mammalian target of rapamycin (Raptor) and proline-rich Akt substrate of 40 kDa (PRAS40), whereas mTORC2 contains mammalian stress-activated map kinase-interacting protein 1 (mSin1), rapamycin-insensitive companion of mTOR (Rictor) and protein observed with Rictor 1 and 2 (protor1/2) [6], [10]–[21]. mTORC1 is rapamycin-sensitive and involved in cell proliferation, cell growth, metabolism, autophagy and so on [2], [6]. Eukaryotic translation initiation factor 4E-binding protein 1 (4E-BP1) and ribosomal protein S6 kinase of 70kDa (p70S6K) are major downstream targets of mTORC1 and play crucial roles in the regulation of protein synthesis [3]. However, mTORC2 is resistant to acute rapamycin treatment and participates in AKT (Ser473) pathway and actin cytoskeleton organization [16], [17].

Although mTOR signaling contributes to many cellular processes, little is known about the regulation of mTOR expression. Until recently, only several microRNAs (such as miR-99a, miR-7, miR-199a-3p, miR-100 and miR-144) are reported to influence the protein level of mTOR possibly by binding its 3′-untranslational region [22]–[29]. Here, we find that TBCK (TBC1 domain containing kinase) influences the expression of mTORC components at the transcriptional level, and is involved in the regulation of cell proliferation, cell growth and actin organization.

## Materials and Methods

### Cloning of Human TBCK and Plasmid Construction

Based on the TBCK sequence in NCBI (GeneID: 93627), the following primers: 5′-GACTCTCGAGTCATGTTTCCCCTGAAGGACGCTG-3′ and 5′-GACTGGATCCACTGTGCTGTTGGTGCTGATGC-3′ were designed to clone TBCK by RT-PCR from total RNA extracted from HEK293 cells.

Full length TBCK cDNA was subcloned into pEGFP-C1 vector. Lentivirus-based RNA interference plasmid construction, lentivirus harvesting, and lentivirus titer testing were made by GENECHEM Company (Shanghai, China). All of these constructs were confirmed by DNA sequencing.

### Anti-TBCK Antibody Production

Rabbit polyclonal anti-TBCK antibody was generated by KLH-conjugated peptide (LFEDGESFGQGRDRSSLLDDT, ProteinTech, Wuhan, China) and was affinity purified.

### Cell Culture, Transfection and Lentivirus Infection

HeLa cells, HEK293 cells, A549 cells, HepG2 cells, and T98G cells were purchased from ATCC. AGS cells and NCI-N87 cells were purchased from the Cell Bank of Chinese Academy of Sciences (Shanghai, China). Cells were maintained in DMEM containing 10% Fetal Bovine Serum (FBS) and transfected by PolyJet transfection reagent (SignaGen Laboratories, Rockville, MD). HEK293 cells were infected with lentivirus of RNAi control, TBCK-RNAi-1 (targeting the region 1299–1317nt relative to the first nucleotide of the start codon, 5′-G GAT ACA GAG TAC CAA CTA-3′), and TBCK-RNAi-2 (targeting the region 2524-2544nt relative to the first nucleotide of the start codon, 5′-GGG AAG GTC ATT GTC ATC GTG-3′) at MOI 25 in the presence of 5 µg/ml polybrene.

### Cell Proliferation Assay

For cell proliferation analysis, lentivirus-infected cells were seeded into 24-well plates at 1×10^4^ cells/well and the cell numbers were determined daily for 4 days. For MTT (3-[4,5-Dimethylthiazol-2-yl]-2,5-diphenyltetrazolium bromide) assay, lentivirus-infected cells were seeded into 96-well plates at 2000 cells/well. On the day of harvest, 100 μl of spent medium was replaced with an equal volume of fresh medium containing 1 mg/ml MTT. The medium was replaced by 150 μl of DMSO after incubation at 37°C for 4 h and plates were shaken at room temperature for 10 min. The absorbance was measured at 570 nm.

### Immunofluorescence Staining

HEK293 cells or HeLa cells were seeded on coverslips and cultured in DMEM with 10% fetal bovine serum. Cells were fixed with 4% formaldehyde diluted in PBS for 15 min at room temperature and then incubated with anti-TBCK antibody, anti-GM130 antibody (BD Biosciences), anti-PDI antibody, anti-MPR antibody (Abcam), anti-p58 antibody, anti-α-tubulin antibody, anti-γ-tubulin antibody (Sigma-Aldrich), anti-mTOR antibody, or anti-phospho-mTOR antibody (Cell Signaling Technology) for 2 h at room temperature, followed by staining with either Alexa Fluor 488- or Alexa Fluor 555-conjugated anti-mouse or anti-rabbit Ig secondary antibody (Invitrogen) for 1 h at room temperature. Finally, DNA was visualized with DAPI (Sigma-Aldrich) and F-actin was stained with phalloidin (Sigma-Aldrich). The mounted coverslips were analyzed with confocal fluorescence microscopy (LSM510 Meta; Carl Zeiss). Cell area was calculated by Image-Pro Plus 6.0 software (Media Cybernetics Inc., Rockville, MD).

### Cell Size Determination

For measurements of the size of unfixed cells, lentivirus-infected HEK293 cells were washed twice with PBS, pelleted, and resuspended in PBS containing 10 μg/ml RNase A and 10 μg/ml propidium iodide (Bio Basic Inc., Markham, Ontario). The samples were subsequently analyzed by fluorescence-activated cell sorters (FACS Calibur; BD Biosciences) for cell size (FSC-H, forward scatter height). PI-positive cells were excluded from the analyses. GFP-positive (lentivirus infected cells), PI-negative cells were gated and analyzed. The mean of FSC-H was determined [30].

### Real Time Quantitative RT-PCR Analysis

Reverse transcription reaction was carried out starting from 1 μg total RNA extracted from HEK293 cells using M-MLV Reverse Transcriptase (Invitrogen). FastStart Universal SYBR Green Master (ROX) (Roche) and CFX96 Touch™ Real-Time PCR Detection System (Bio-Rad, Shanghai, China) were used for a real-time PCR and data were analyzed with Bio-Rad CFX Manager (V1.5.534.0511). The ΔΔCt method for relative quantization was used to determine mRNA expression. Fold change was determined as 2^−ΔΔCt^ and target mRNA expression was normalized to GAPDH.

### Statistical analyses

Data are reported as mean ± SE. The significance of the observed differences was determined by Student's t-test. *P*<0.05 was considered to be statistically significant.

## Results

### Identification of TBCK protein

**Figure 1:**
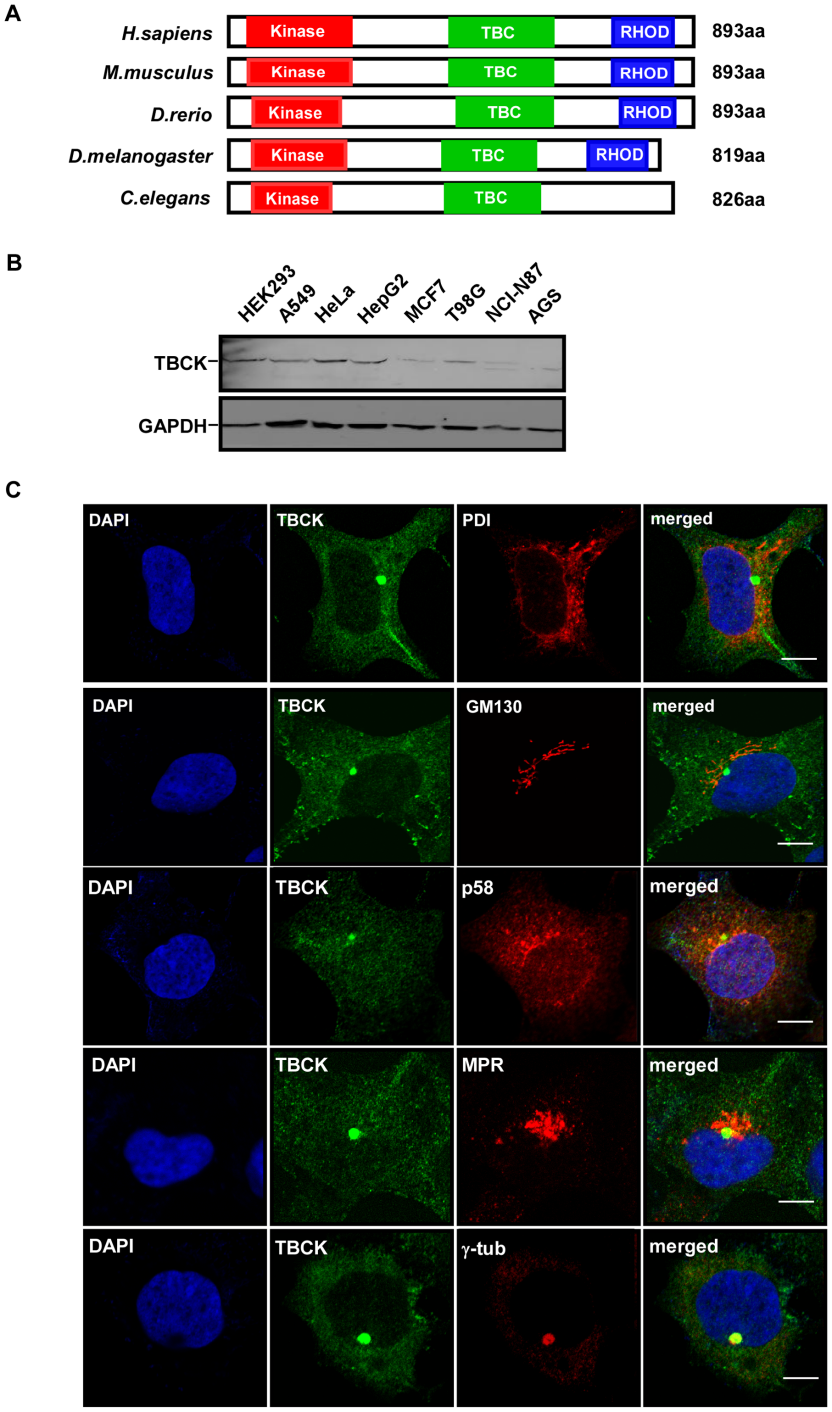
Characterization of TBCK. (**A**) Schematic comparison of TBCK homologs in *H. sapiens*, *M. musculus*, *D. rerio*, *D. melanogaster*, and *C. elegans*. Red, green and blue bars indicate possible kinase domain, TBC domain and rhodanese homology domain, respectively. (**B**) Expression of TBCK in mammalian cell lines. Total lysates from the indicated cells were resolved by SDS-PAGE and subjected to Western analysis. (**C**) Subcellular localization of TBCK. HEK293 cells grown on cover slides were immunostained with the antibodies as shown. DNA was visualized by DAPI. Bar, 10 μm.

To discover novel protein kinases, we used tBLASTn program to search uncharacterized proteins with kinase domains and found a putative conserved protein TBCK (NP_001156908) that contains a likely kinase domain, TBC (Tre-2/Bub2/Cdc16) domain and rhodanese homology domain (RHOD). Human TBCK cDNA was cloned by RT-PCR with total RNA extracted from HEK293 cells (Fig. S1). Sequencing analysis showed that TBCK (NM_001163436) encoded a deduced 893-aa protein with a predicted molecular mass of 99 kDa. BLAST searches for proteins homologous to human TBCK in other species revealed that putative orthologs of TBCK are highly conserved in *C. elegans*, *D. melanogaster*, *D. rerio* and *M. musculus* (Fig. 1A).

To explore the expression and subcellular localization of TBCK, we raised a rabbit polyclonal antibody against a synthetic peptide of TBCK (Fig. S2A). After affinity purification, the anti-TBCK antibody specifically recognized endogenous TBCK protein by Western blotting and immunofluorescence microscopy (Fig. S2B and S2C). Immunoblot analysis showed that TBCK is ubiquitously expressed in different cell lines, including HEK293 cells, A549 cells, HeLa cells, HepG2 cells, MCF7 cells, T98G cells, NCI-N87 cells and AGS cells (Fig. 1B). Immunofluorescence analysis revealed that TBCK was clearly colocalized with γ-tubulin in addition to punctate distribution in HEK293 cells (Fig. 1C). However, we observed that TBCK was usually located near the nucleus and around centrosomes, but not merged with γ-tubulin in HeLa cells (Fig. S3). TBCK appeared to be not substantially colocalized with endoplasmic reticulum marker PDI (Protein Disulfide Isomerase), Golgi markers GM130 (*cis*-Golgi matrix protein of 130 kDa) and p58 (Golgi 58K protein), and late endosome marker MPR (Mannose 6 Phosphate Receptor) in both HEK293 and HeLa cells (Fig. 1C and Fig. S3).

### Depletion of TBCK inhibits cell proliferation and cell size

**Figure 2:**
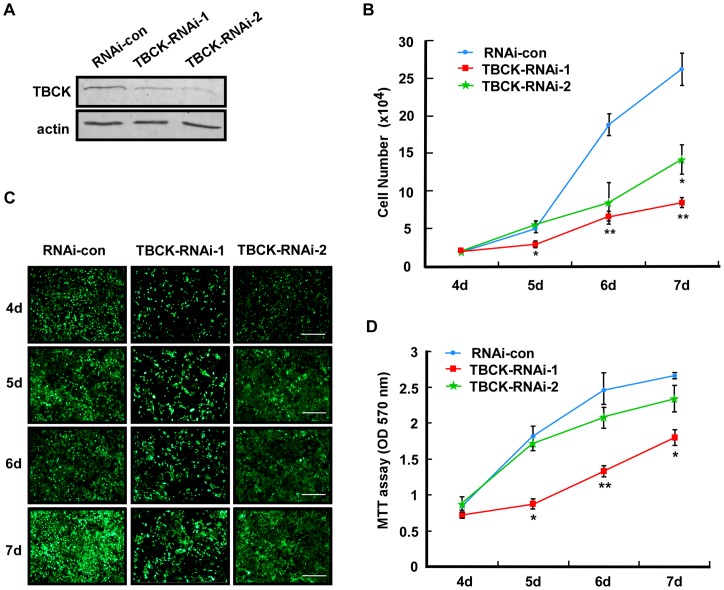
Depletion of TBCK inhibits cell proliferation. HEK293 cells infected with the lentiviruses targeting to the various regions of TBCK mRNA (TBCK-RNAi-1 or 2) were subjected to Western blotting (**A**), cell counts (**B**), fluorescence microscopy (**C**) and MTT analyses (**D**). Bar, 200 µm. Lentivirus-infected cells were GFP-positive. Data are shown as the mean of three independent experiments ± SE (* *P*<0.05, ** *P*<0.01).

To investigate the function of TBCK, we depleted endogenous TBCK by lentivirus-based RNAi targeting to two different regions of TBCK mRNA (TBCK-RNAi-1 and TBCK-RNAi-2). Western blotting showed that the protein level of TBCK was decreased in cells infected with either TBCK-RNAi-1 or TBCK-RNAi-2 compared with that of control virus infection (RNAi-con) (Fig. 2A). Cell count, fluorescence microscopy and MTT analyses revealed that TBCK depletion effectively inhibited the proliferation of HEK293 cells (Figs. 2B–2D).

**Figure 3:**
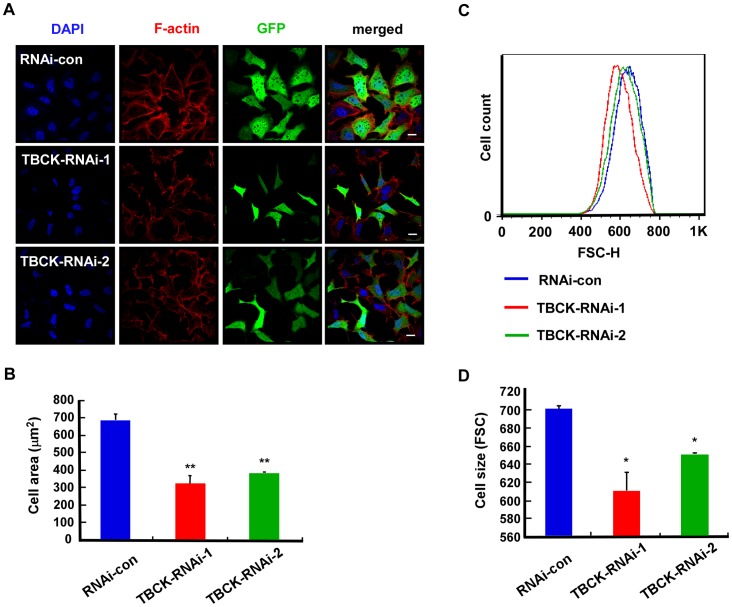
TBCK depletion reduces cell size. HEK293 cells were infected with the indicated lentivirus and subjected to fluorescence microscopy (A–B) and FACS analysis (C–D). (**A**–**B**) GFP-positive signals indicate the cells infected by lentivirus. Bar, 20 µm. The area measurements of infected cells were quantified by Image-Pro Plus 6.0 software. Data are shown as the mean of three independent experiments ± SE (** *P*<0.01, n>200). (**C**–**D**) Representative histogram of flow cytometry shows the size distribution (FSC-H) of GFP positive cells that were stained with propidium iodide. The mean of three independent experiments ± SE is shown (* *P*<0.05, n>100,000).

Unexpectedly, we found that the size of TBCK-depleted cells were obviously decreased compared with that of the control cells (Fig. 3A), which was confirmed by Image-Pro Plus software analysis (Fig. 3B). To further examine the role of TBCK in the regulation of cell size, we employed flow cytometer to measure the mean of cell sizes (more than 100,000 cells) and found that depletion of TBCK significantly reduced cell size (Fig. 3C and 3D). Thus, these results indicate that TBCK may be involved in cell proliferation and cell growth.

### TBCK plays a role in actin organization

**Figure 4:**
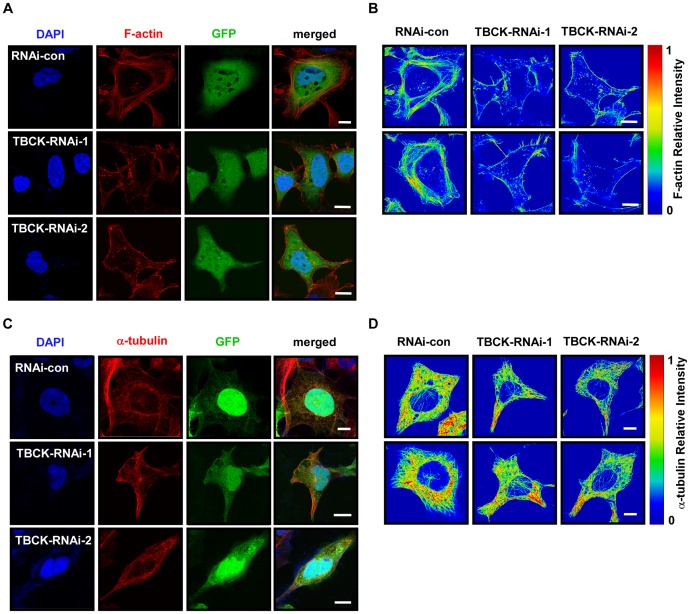
TBCK plays a role in actin organization. HEK293 cells infected with the indicated lentivirus were subjected to immunofluorescence staining with phalloidin-TRITC (**A**) or anti-α-tubulin antibody (**C**). Lentivirus-infected cells were GFP-positive. DNA was visualized by DAPI. Bar, 10 µm. Single panel image of phalloidin-TRITC signal (**B**) or α-tubulin (**D**) is showed in rainbow palettes.

Because the cell size is closely related to cytoskeleton organization, we examined the effect of TBCK on the regulation of actin and microtubule. TBCK depletion induced a robust decrease in F-actin and disrupted stress fibers, whereas cortical actin appeared to be not affected, which is validated by measuring relative intensity of F-actin using rainbow palette (Fig. 4A and 4B). However, depletion of TBCK did not considerably alter microtubule structure compared with the control cells (Fig. 4C and 4D). These data suggest that TBCK may be involved in the organization of actin, but not microtubule.

### TBCK Depletion influences mTOR signaling pathway

**Figure 5:**
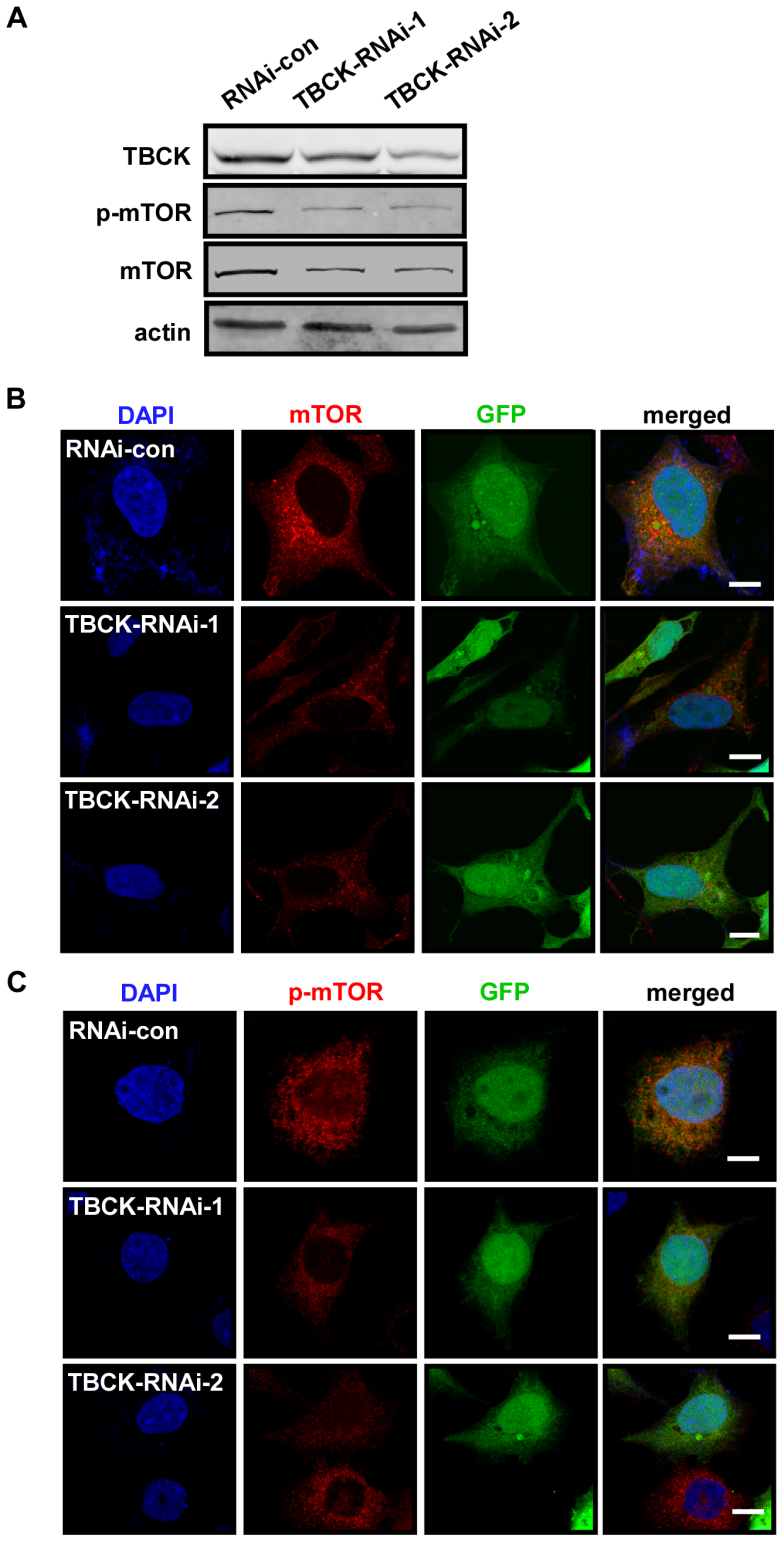
Depletion of TBCK influences mTOR signaling pathway. HEK293 cells were infected with the indicated lentivirus and subjected to Western blotting (**A**) and immunofluorescence analysis (**B**–**C**) with the antibodies as shown. GFP-positive signals indicate the cells infected by lentivirus. DNA was visualized by DAPI. Bar, 10 µm.

**Figure 6:**
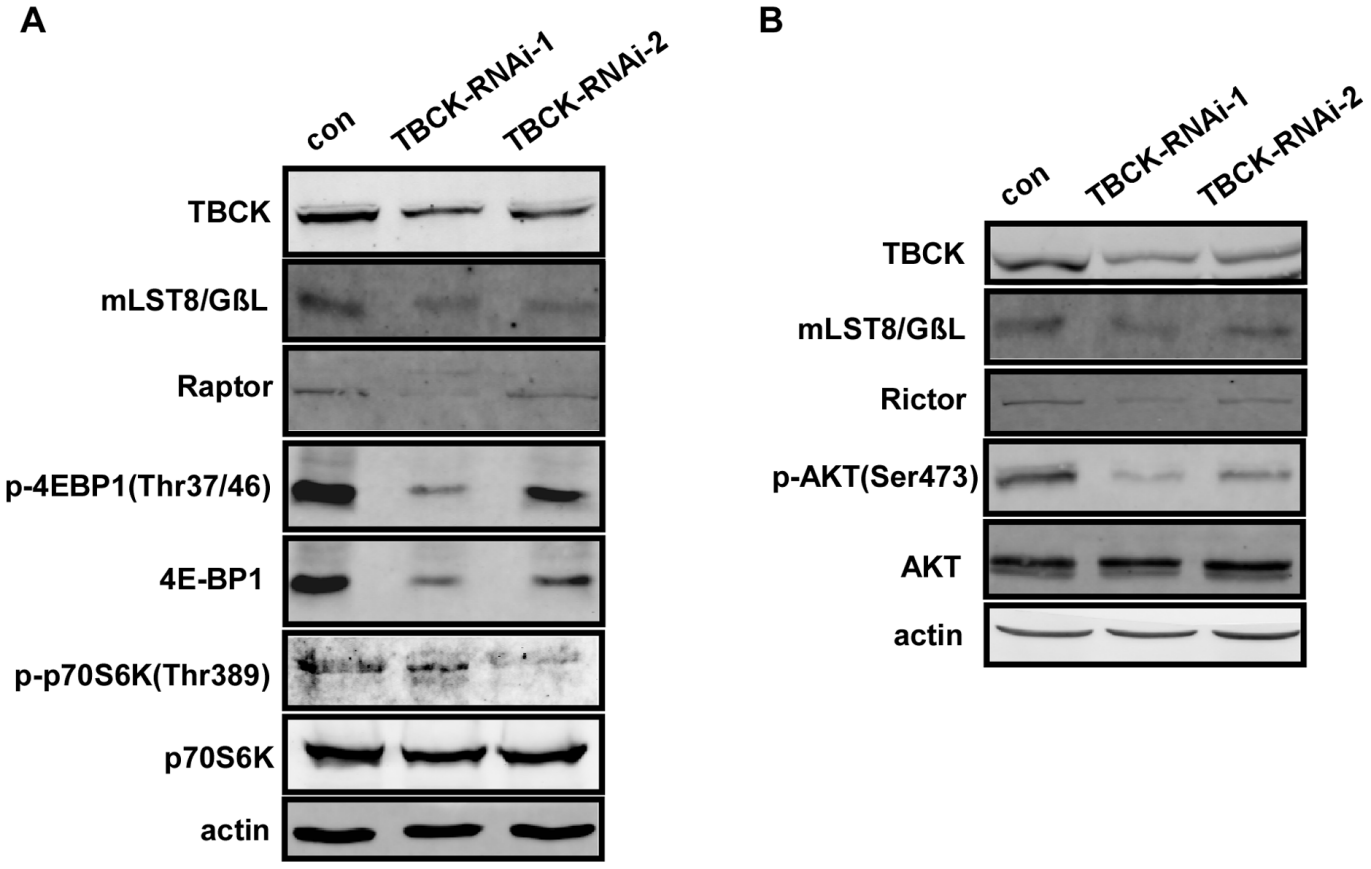
TBCK influences the protein level of mTOR components and the activities of mTOR complexes. (**A**–**B**) HEK293 cells infected with the indicated lentivirus were subjected to immunoblotting with the antibodies as shown.

Since mTOR pathway plays a key role in regulating cell proliferation, cell growth and actin organization [6], we reasoned that TBCK may regulate mTOR pathway. Western analysis showed that knockdown of TBCK decreased the protein level and phosphorylation of mTOR (Fig. 5A), which is supported by immunofluorescence analysis (Fig. 5B and 5C). Further experiments revealed that depletion of TBCK also reduced the protein level of mTORC components, including Raptor, Rictor and mLST8/GβL (Fig. 6A and 6B). The phosphorylation of downstream substrates of mTORC, including 4E-BP1 (Thr37/46), p70S6K (Thr389) and Akt (Ser473), was substantially inhibited in cells depleted of TBCK. Interestingly, the protein level of 4E-BP1, but not p70S6K and Akt, was also downregulated in TBCK-depleted cells (Fig. 6A and 6B). Taken together, these results imply that TBCK may modulate mTOR signaling pathway by influencing mTOR signaling components.

**Figure 7:**
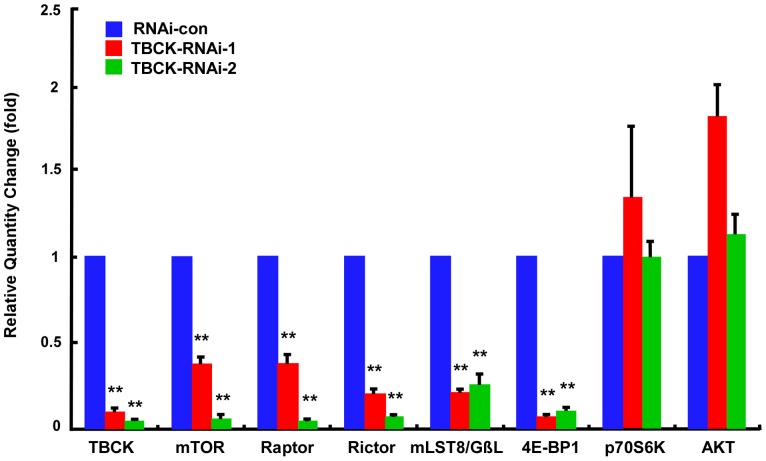
Depletion of TBCK represses the mRNA level of mTOR complexes components. Total RNAs from HEK293 cells infected with the indicated lentivirus were analyzed by quantitative RT-PCR with the targeted genes as shown. Error bars indicate SE (** *P*<0.01).

To further evaluate how TBCK affects the protein level of mTOR signaling constituents, we did quantitative real-time PCR (qRT-PCR) analyses and found that knockdown of TBCK significantly repressed the expression of mTORC components including mTOR, Raptor, Rictor and mLST8/GβL, and its downstream substrate 4E-BP1 (*P*<0.01), but not p70S6K and Akt (*P*>0.05) (Fig. 7). These data suggest that TBCK is involved in mTOR signaling pathway at the level of transcription.

**Figure 8:**
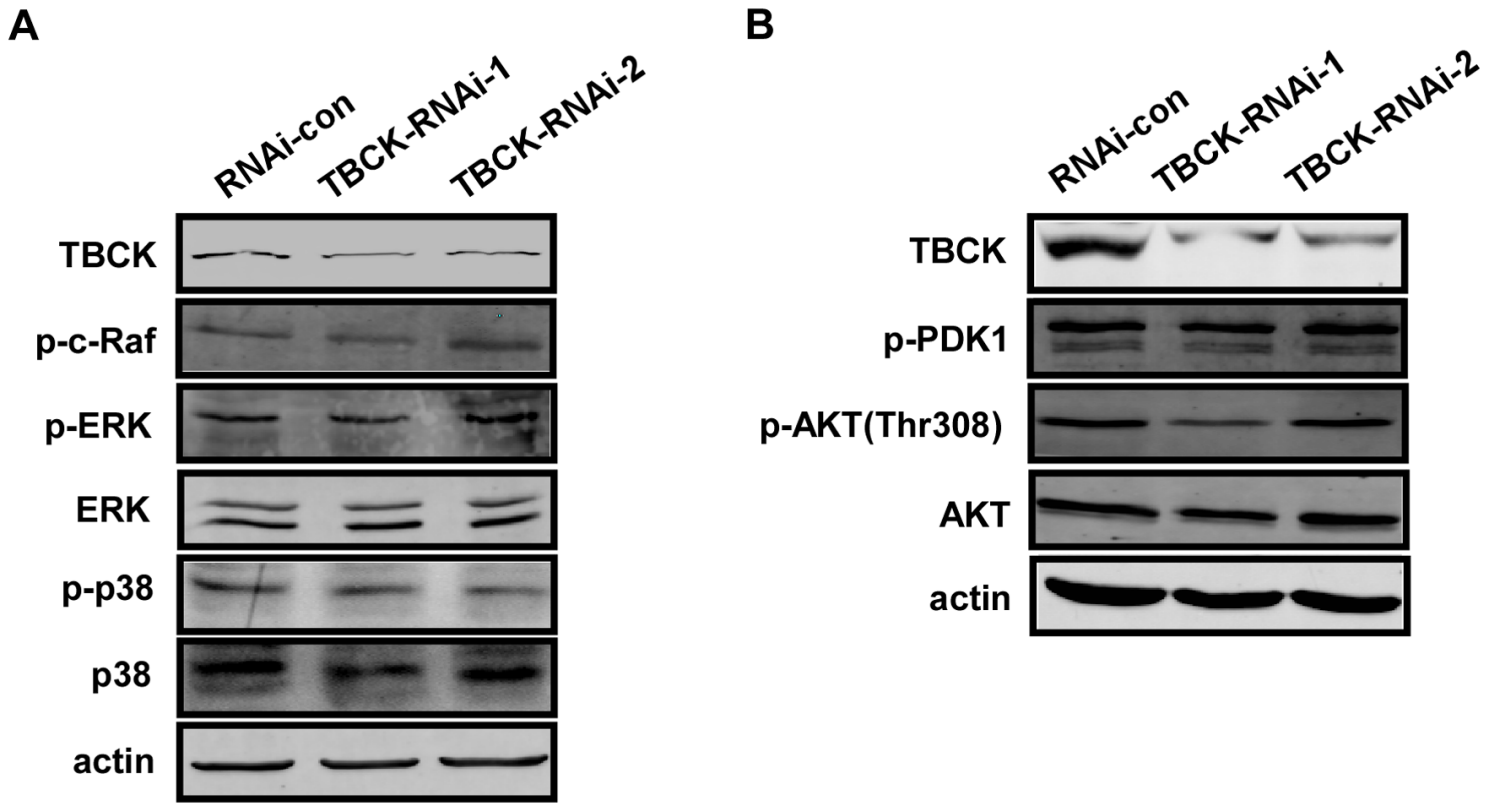
TBCK has no significant effect on MAPK and PDK1/AKT pathway. (**A**–**B**) HEK293 cells infected with the indicated lentivirus were subjected to Western blotting with the antibodies as shown.

To determine whether the role of TBCK in mTOR signaling pathway is specific or not, we analyzed the effect of TBCK on MAPK and PDK1/AKT pathways. The results showed that TBCK depletion did not influence the phosphorylation of c-Raf, ERK, p38MAPK (Fig. 8A), PDK1 and Akt (Thr308) (Fig. 8B).

## Discussion

TBCK was first annotated in kinome study and has not been systematically characterized until now [31]. A genome-wide screening project for cancer mutation has found that there are one missense mutation and one frameshift deletion of TBCK in colorectal adenocarcinoma and one missense mutation in head and neck squamous cell carcinoma [32]. Here, we provide evidence that depletion of TBCK significantly inhibits cell proliferation, reduces cell size, and disrupts the organization of actin. Further results show that knockdown of TBCK induces a significant decrease in the protein and mRNA levels of mTORC components. These data suggest that TBCK may regulate cell proliferation, cell growth and actin organization possibly by enhancing mTOR pathway.

Bioinformatics analysis indicates that human TBCK possesses three putative domains: kinase domain, TBC domain and RHOD domain. In general, kinase domain has 12 conserved structural subdomains required for kinase activity [33]. The G-loop (GxGxxG motif) in subdomain I and the VAIK motif in subdomain II that are responsible for ATP binding are degraded in TBCK (Fig. S4). The aspartate residue of HRD motif in subdomain VIb essential for catalytic activity has been replaced by alanine in TBCK protein. In TBCK, the aspartate residue of DFG motif in subdomain VII that is critical for metal binding in *bona fide* kinase has been substituted by lysine. Thus, TBCK has been assumed as a pseudokinase [34], [35]. Further investigation is clearly needed to carefully determine whether TBCK has kinase activity.

Rabs are GTP-binding proteins with conserved functions in membrane trafficking including vesicle budding, vesicle tethering and membrane fusion [36]–[38]. A number of TBC domain-containing proteins have been characterized as RabGAP (Rab GTPase-activating protein) that catalyzes GTP hydrolysis of Rab proteins [39]. The TBC domain in TBCK has the key conserved amino acid residues required for RabGAP activity in functional RabGAPs [40] (Fig. S5). However, a systematical screening for target Rabs of TBC domain-containing proteins (40 proteins including TBCK) based on their Rab-binding activity was failed to find the paired Rab protein for TBCK [41]. Thus, an important future study of TBCK is to identify the physiological target Rab of TBCK.

Emerging data show that TBC domain-containing proteins may be involved in mTOR signaling pathway. *TSC1* and *TSC2* are the tumor-suppressor genes mutated in the tumor syndrome TSC (tuberous sclerosis complex) and their gene products form a complex (TSC1–TSC2) that integrates signals upstream of mTOR signaling pathway through its GAP activity towards the small G-protein Rheb [42]. Recently, TBC1D7 (TBC domain family, member 7) has been found to be a third functional component of the TSC1–TSC2 complex and regulate mTORC1 pathway possibly through their Rheb-GAP activity [43]–[45]. TBC1D7 depletion increases mTORC1 signaling, delays induction of autophagy, and enhances cell growth under poor growth conditions [43].

In this study, we found that TBCK knockdown significantly inhibited mTOR signaling and the expression of mTOR, Raptor, Rictor, mLST8/GβL and 4E-BP1; however, the underlying mechanism is still unknown. One possibility is that TBCK may be involved in the transcriptional regulation of mTORC constituents. We used *cis*-regulatory elements database (cisRED) to analyze the putative transcription factors that may bind to the upstream region of mTORC component genes [46], but failed to find any common transcription factors that may target to all of these mTORC constituents (Table S1). Alternatively, TBCK may influence the mRNAs of mTORC constituents at post-transcriptional level. The 5′- and 3′- untranslated regions (5′-UTR and 3′-UTR) of mRNA are essential for post-transcriptional regulation of gene expression including mRNA stability and translation [47]. We employed UTRscan software and found several conserved mRNA regulators that are predicted to associate with the 5′-UTR or 3′-UTR of mTOR components [48] (Table S2). One of them is Musashi protein that is able to associated with Musashi binding element (MBE) to influence the stability and translation of target mRNAs [49], [50]. Further functional characterization of TBCK will provide important insights into the regulation of mTOR pathway.

RHOD domain is found in rhodanese and rhodanese domain-containing proteins including the Cdc25 class of protein phosphatases [51], [52]. Rhodanese that catalyzes the transfer of a sulfane sulfur atom from thiosulfate to cyanide is important for cyanide detoxification [51]. It will be interesting to examine whether TBC domain or RHOD domain is involved in regulating the expression of mTORC components.

In conclusion, we show for the first time that TBCK is involved in the regulation of cell proliferation, cell growth and actin organization probably by modulating mTOR pathway.

## Supporting Information

Figure S1 **Molecular cloning of human TBCK gene.** According to human TBCK sequence in NCBI (Gene ID: 93627), we designed forward primer 5′-GACTCTCGAGTCATGTTTCCCCTGAAGGACGCTG-3′ and reverse primer 5′-GACTGGATCCACTGTGCTGTTGGTGCTGATGC-3′ to clone TBCK from total RNA extracted from HEK293 by RT-PCR (line 2). The molecular marker of DNA is shown (line 1).
(TIF)

Figure S2 **Characterization of affinity-purified anti-TBCK polyclonal antibody.** (**A**) The peptide sequence from Human TBCK (359–379 aa) for antibody production is marked in red. (**B**) Identification of anti-TBCK peptide antibody. Rabbit polyclonal antibody against TBCK was generated by using the synthetic KLH (Keyhole limpet hemocyanin)-conjugated peptide of TBCK as antigen and then affinity-purified with this synthetic TBCK peptide. Total lysates from HEK293 cells transfected with either EGFP-C1 vector or EGFP-C1-TBCK were subjected to Western analysis with anti-TBCK antibody with various amounts of the synthetic TBCK peptide. (**C**) HeLa cells grown on cover slides were immunostained with the indicated antibodies in the present or absent of synthetic TBCK peptide. DNA was visualized by DAPI. Bar, 10 μm.
(TIF)

Figure S3 **Subcellular localization of TBCK in HeLa cells.** HeLa cells grown on cover slides were subjected to immunofluorescence analyses with the indicated antibodies. DNA was stained by DAPI. Bar, 10 µm.
(TIF)

Figure S4 **Sequence analysis of the potential kinase domain of TBCK.** ClustalW2 algorithm (http://www.ebi.ac.uk/Tools/msa/clustalw2/) was used to compare the kinase domains of human STK24 (serine/threonine-protein kinase 24, NP_001027467), MST1 (mammalian ste20-like kinase 1, AAA83254), GCK (Germinal center kinase, NP_004570), CDK2 (cyclin-dependent kinase 2, CAA43807), PAK1 (p21 protein-activated kinase 1, AAC50590) and TBCK. The subdomains of kinase domains are boxed by black lines. The red boxes indicate the key amino acid residues in the kinase domain of TBCK different with other *bona fide* kinases.
(TIF)

Figure S5 **Sequence analysis of the TBC domain of TBCK.** ClustalW2 algorithm (http://www.ebi.ac.uk/Tools/msa/clustalw2/) was employed to compare the TBC domains of hRN-tre (Related to the N-terminus of tre, NP_055503), AS160/TBC1D4 (Akt substrate of 160 kDa/TBC1 domain family member 4, NP_055647), Gyp1p (yeast Rab GTPase-activating protein, NP_014713), Bub2p (yeast Bub2p spindle checkpoint protein, NP_013771) and TBCK. The conserved amino acids responsible for RabGAP activity are highlighted by red rectangles.
(TIF)

Table S1 **The putative transcription factors of mTORC components.**
(XLS)

Table S2 **The predicted regulatory elements of mTORC component mRNAs.**
(XLS)
