# Supplementary figures and images for: TBCK Influences Cell Proliferation, Cell Size and mTOR Signaling Pathway
Source: PLoS One. 2013 Aug 19;8(8):e71349. doi: 10.1371/journal.pone.0071349 (PMC3747267; doi:10.1371/journal.pone.0071349)

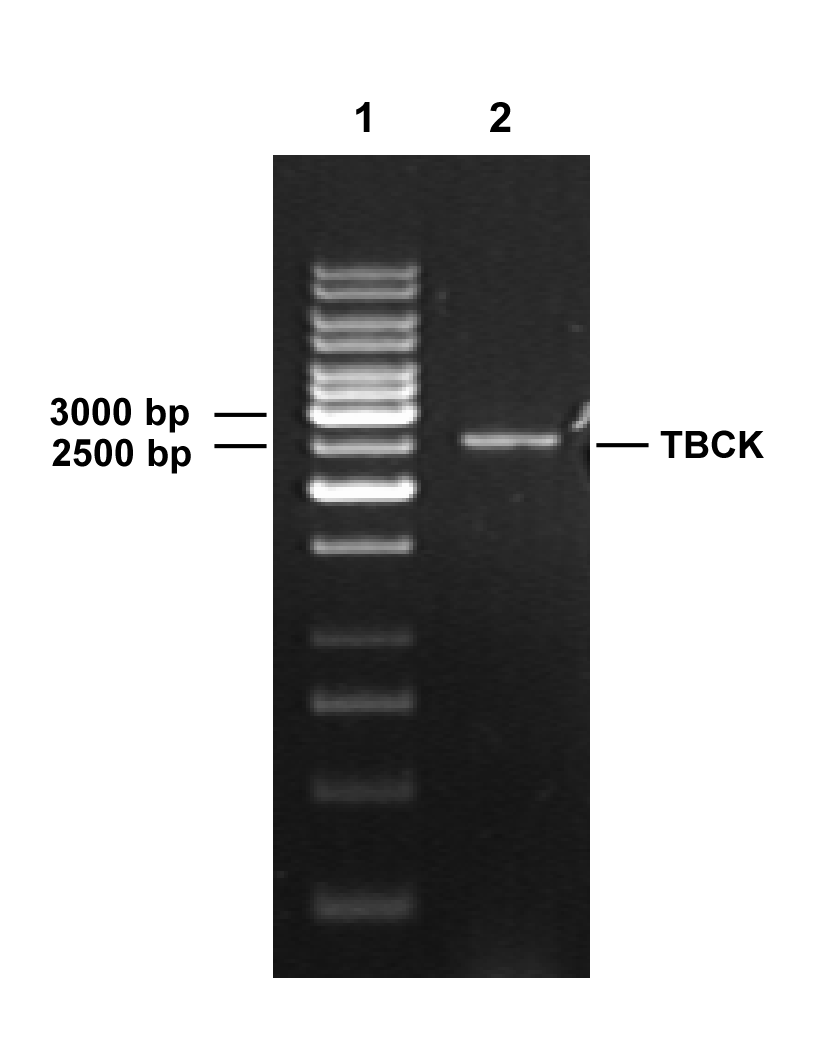

Supplement: Figure S1 — Molecular cloning of human TBCK gene. According to human TBCK sequence in NCBI (Gene ID: 93627), we designed forward primer 5′-GACTCTCGAGTCATGTTTCCCCTGAAGGACGCTG-3′ and reverse primer 5′-GACTGGATCCACTGTGCTGTTGGTGCTGATGC-3′ to clone TBCK from total RNA extracted from HEK293 by RT-PCR (line 2). The molecular marker of DNA is shown (line 1). (TIF) [file pone.0071349.s001.tif]

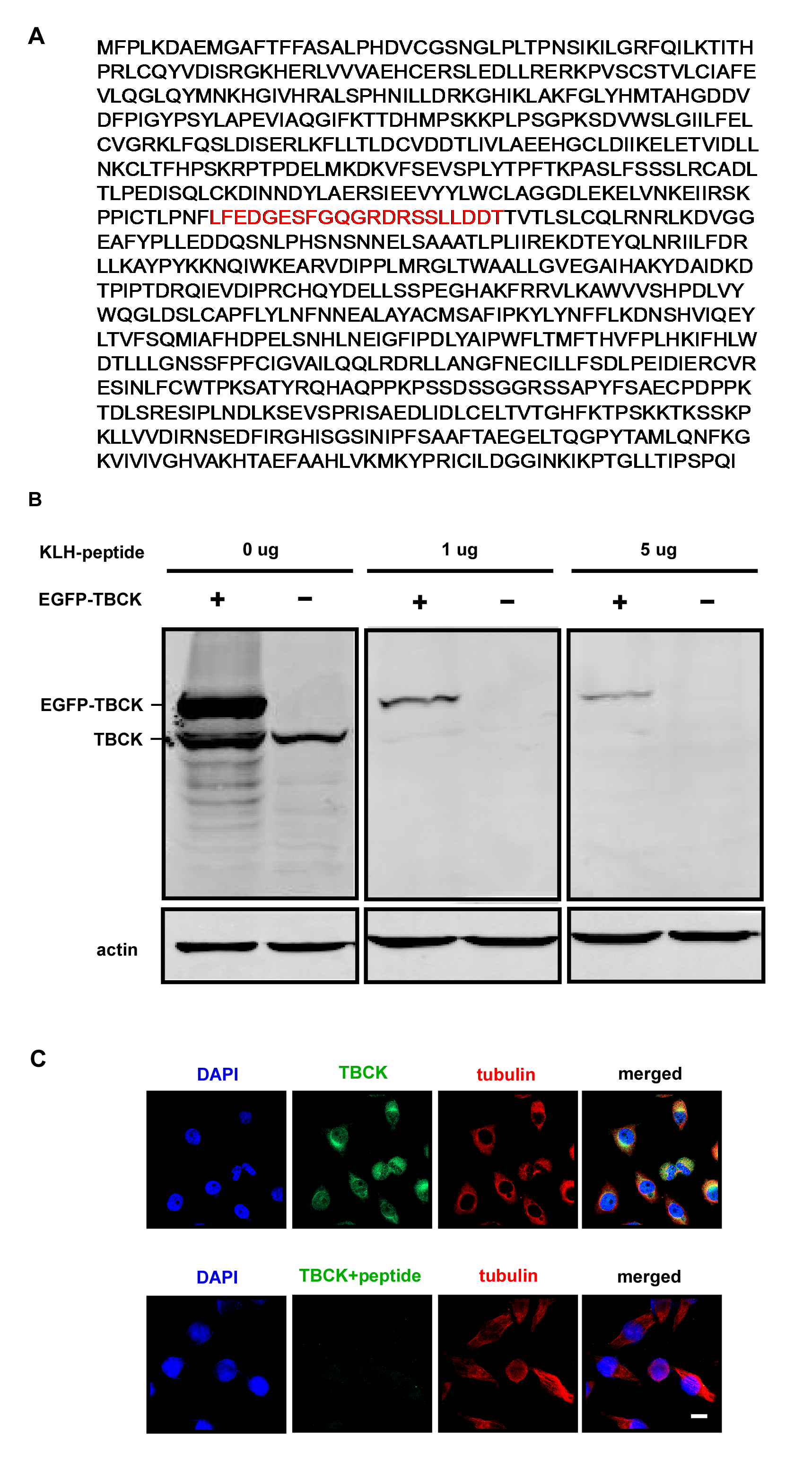

Supplement: Figure S2 — Characterization of affinity-purified anti-TBCK polyclonal antibody. (A) The peptide sequence from Human TBCK (359–379 aa) for antibody production is marked in red. (B) Identification of anti-TBCK peptide antibody. Rabbit polyclonal antibody against TBCK was generated by using the synthetic KLH (Keyhole limpet hemocyanin)-conjugated peptide of TBCK as antigen and then affinity-purified with this synthetic TBCK peptide. Total lysates from HEK293 cells transfected with either EGFP-C1 vector or EGFP-C1-TBCK were subjected to Western analysis with anti-TBCK antibody with various amounts of the synthetic TBCK peptide. (C) HeLa cells grown on cover slides were immunostained with the indicated antibodies in the present or absent of synthetic TBCK peptide. DNA was visualized by DAPI. Bar, 10 μm. (TIF) [file pone.0071349.s002.tif]

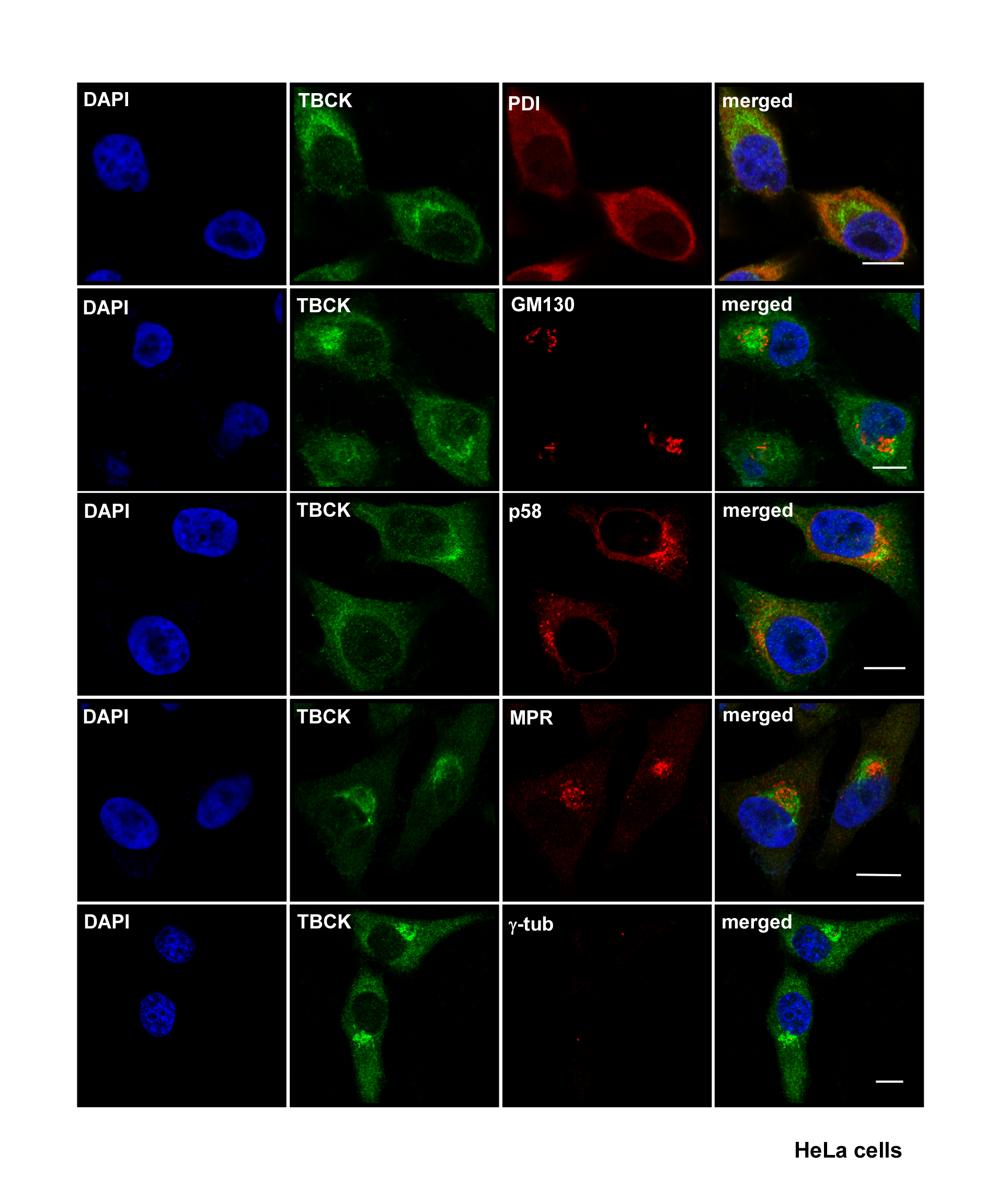

Supplement: Figure S3 — Subcellular localization of TBCK in HeLa cells. HeLa cells grown on cover slides were subjected to immunofluorescence analyses with the indicated antibodies. DNA was stained by DAPI. Bar, 10 µm. (TIF) [file pone.0071349.s003.tif]

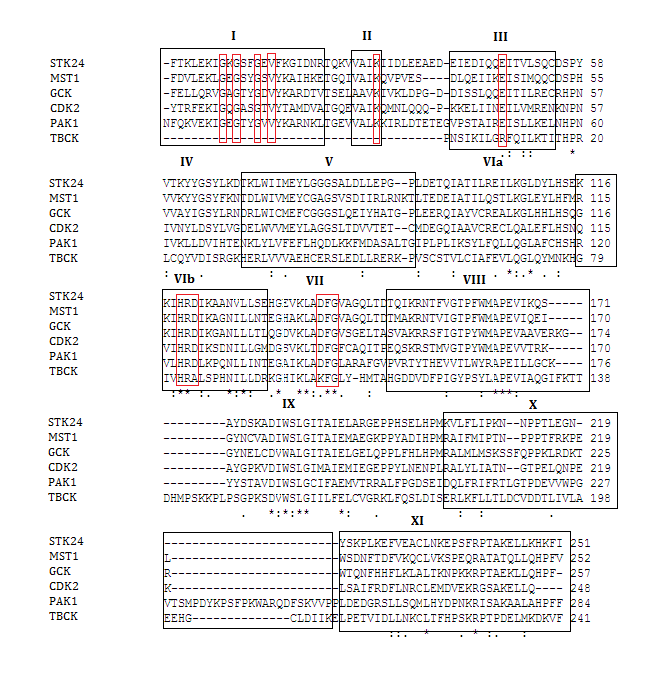

Supplement: Figure S4 — Sequence analysis of the potential kinase domain of TBCK. ClustalW2 algorithm (http://www.ebi.ac.uk/Tools/msa/clustalw2/) was used to compare the kinase domains of human STK24 (serine/threonine-protein kinase 24, NP_001027467), MST1 (mammalian ste20-like kinase 1, AAA83254), GCK (Germinal center kinase, NP_004570), CDK2 (cyclin-dependent kinase 2, CAA43807), PAK1 (p21 protein-activated kinase 1, AAC50590) and TBCK. The subdomains of kinase domains are boxed by black lines. The red boxes indicate the key amino acid residues in the kinase domain of TBCK different with other bona fide kinases. (TIF) [file pone.0071349.s004.tif]

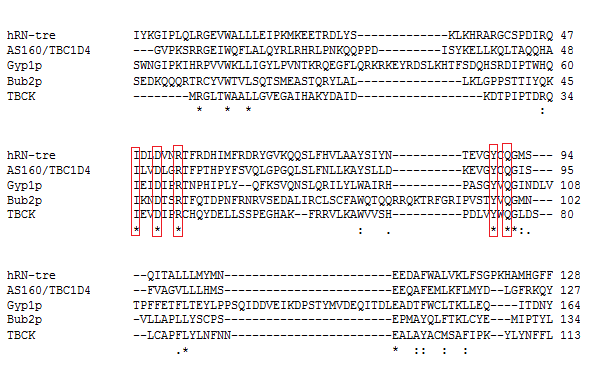

Supplement: Figure S5 — Sequence analysis of the TBC domain of TBCK. ClustalW2 algorithm (http://www.ebi.ac.uk/Tools/msa/clustalw2/) was employed to compare the TBC domains of hRN-tre (Related to the N-terminus of tre, NP_055503), AS160/TBC1D4 (Akt substrate of 160 kDa/TBC1 domain family member 4, NP_055647), Gyp1p (yeast Rab GTPase-activating protein, NP_014713), Bub2p (yeast Bub2p spindle checkpoint protein, NP_013771) and TBCK. The conserved amino acids responsible for RabGAP activity are highlighted by red rectangles. (TIF) [file pone.0071349.s005.tif]
